# A scoping review of physiological biomarkers in autism

**DOI:** 10.3389/fnins.2023.1269880

**Published:** 2023-09-07

**Authors:** Jiatong Shan, Yunhao Gu, Jie Zhang, Xiaoqing Hu, Haiyan Wu, Tifei Yuan, Di Zhao

**Affiliations:** ^1^Shanghai Key Laboratory of Psychotic Disorders, Brain Health Institute, National Center for Mental Disorders, Shanghai Mental Health Center, Shanghai Jiao Tong University School of Medicine, Shanghai, China; ^2^Department of Arts and Sciences, New York University Shanghai, Shanghai, China; ^3^Graduate School of Education, University of Pennsylvania, Philadelphia, PA, United States; ^4^Department of Neurology, Institute of Neurology, Sichuan Provincial People’s Hospital, School of Medicine, University of Electronic Science and Technology of China, Chengdu, China; ^5^Department of Psychology, The State Key Laboratory of Brain and Cognitive Sciences, The University of Hong Kong, Hong Kong, Hong Kong SAR, China; ^6^HKU, Shenzhen Institute of Research and Innovation, Shenzhen, China; ^7^Center for Cognitive and Brain Sciences and Department of Psychology, Macau, China

**Keywords:** autism spectrum disorder, physiological biomarkers, electroencephalography, magnetoencephalography, electrodermal activity markers, eye-tracking markers, neurodevelopmental deficits

## Abstract

Autism spectrum disorder (ASD) is a neurodevelopmental condition characterized by pervasive deficits in social interaction, communication impairments, and the presence of restricted and repetitive behaviors. This complex disorder is a significant public health concern due to its escalating incidence and detrimental impact on quality of life. Currently, extensive investigations are underway to identify prospective susceptibility or predictive biomarkers, employing a physiological biomarker-based framework. However, knowledge regarding physiological biomarkers in relation to Autism is sparse. We performed a scoping review to explore putative changes in physiological activities associated with behaviors in individuals with Autism. We identified studies published between January 2000 and June 2023 from online databases, and searched keywords included electroencephalography (EEG), magnetoencephalography (MEG), electrodermal activity markers (EDA), eye-tracking markers. We specifically detected social-related symptoms such as impaired social communication in ASD patients. Our results indicated that the EEG/ERP N170 signal has undergone the most rigorous testing as a potential biomarker, showing promise in identifying subgroups within ASD and displaying potential as an indicator of treatment response. By gathering current data from various physiological biomarkers, we can obtain a comprehensive understanding of the physiological profiles of individuals with ASD, offering potential for subgrouping and targeted intervention strategies.

## 1. Introduction

### 1.1. Definition and overview of autism spectrum disorder

Autism spectrum disorder (ASD) represents a neurodevelopmental condition characterized by pervasive deficits in social interaction, communication impairments, and the presence of restricted and repetitive behaviors. This intricate disorder impacts individuals across a wide spectrum, exhibiting varying degrees of severity and manifestations. According to the World Health Organization, it is estimated that the median prevalence of ASD children in studies from 2012 to 2021 worldwide is about 1 in 100 children, with a trend of increasing prevalence over time (Zeidan et al., 2022), and however, the 1% prevalence may still underestimate the prevalence in low- and middle-income countries (Elsabbagh et al., 2012). And in Asia, ASD is probably more common than previously thought. The average prevalence of ASD before 1980 was around 1.9/10,000 while in China the median prevalence of ASD among only 2–6-year-old children who are reported from 2000 upwards was 10.3/10,000 (Sun and Allison, 2010). Fundamentally, ASD involves aberrant patterns of brain development and functioning. Neuroscientific investigations have shed light on the underlying neural mechanisms associated with ASD, revealing alterations in brain connectivity, structural anomalies, and disruptions in neurotransmitter systems.

Impaired social interaction stands out as a prominent feature of ASD. Individuals with ASD commonly encounter difficulties in comprehending and responding to social cues, including facial expressions and gestures (Lord et al., 2018). Challenges in establishing and maintaining reciprocal relationships, coupled with deficits in empathetic understanding, further contribute to the observed social impairments in this disorder. Communication deficits also feature prominently in ASD. Language development may be delayed or absent in certain individuals, while others exhibit atypical speech patterns, such as repetitive or idiosyncratic language usage (Mitchell et al., 2006). Difficulties in understanding and employing nonverbal communication, encompassing gestures and body language, are also prevalent among individuals with ASD. Restricted and repetitive behaviors serve as defining characteristics of ASD (Harrop et al., 2014). These behaviors manifest in diverse forms, including repetitive movements, insistence on sameness, highly focused interests, and adherence to routines. Sensory sensitivities, ranging from hypersensitivity to hyposensitivity to sensory stimuli, are frequently observed in individuals with ASD.

The etiology of ASD is multifactorial, encompassing genetic and environmental factors. Advancements in genetic research have identified numerous genes associated with ASD, contributing to our understanding of the underlying biological mechanisms. Additionally, prenatal, and perinatal factors, such as maternal immune activation and exposure to environmental toxins, have been implicated in ASD development.

### 1.2. Significance of investigating physiological biomarkers in ASD research

The absence of a discernible biological signature for ASD, most likely attributable to its inherent heterogeneity, poses challenges in accurate prognosis, including the prediction of treatment response and even diagnosis, thereby complicating the clinical landscape (Shen et al., 2020). The field of ASD faces challenges due to the lack of robust, reliable, and valid biomarkers that can facilitate objective diagnosis and personalized treatment recommendations for patients. In this review, we examine and assess the evidence supporting the most promising biomarkers in ASD. The candidate biomarkers under scrutiny encompass electroencephalography markers (EEG), magnetoencephalography markers (MEG), electrodermal activity markers (EDA), and eye-tracking markers. Our aim is to provide a scoping review of the prevalent views on abnormal physiological behaviors in individuals with ASD.

## 2. Methods

In this study, we conducted a scoping review using Google Scholar as well as PubMed with specific keywords. Subsequently, we implemented three rounds of meticulous screening to identify relevant studies. First, we got 1,544 records from January 2000 to June 2023 by searching relative keywords on PubMed and Google Scholar, and we removed 1,377 records because of duplication. Then we included 81 reports out of 167 records according our inclusion criteria: (1) utilization of a neurophysiological measure; (2) inclusion of an ASD group, encompassing individuals diagnosed with autism, ASD, Asperger syndrome, autistic disorder, or pervasive developmental disorder – not otherwise specified (PDD-NOS); (3) presence of a typically developing (TD) control group; (4) publication in English; and (5) peer-reviewed status. In the third screening process, we examined again based on the previous criteria, and further excluded 31 records further (8 records did not include ASD subjects, 12 records were review, 8 records were non-relevant studies, 1 record was animal model, and 2 were duplicate records). Finally, 50 records were included and analyzed in this scoping review. This whole screening process was done by Jiatong Shan and Di Zhao separately and decision was moderated if there is a difference. As the third party, Yunhao rated each record included, and all included records have relatively high quality.

## 3. Results

### 3.1. Event-related potentials in ASD and its connection to abnormal sensory perception

Researchers employ event-related potentials (ERPs) to assess the processing of sensory stimuli, including social cues. No matter for N1, P1, MMN or P300 waves, the experimenters did not get a unified conclusion on the rules of ASD’s abnormal amplitude and latency. Some studies think that ASD patients are insensitive to stimuli, that is, the amplitude decreases and the latency increases; some studies show that ASD patients are too sensitive to stimuli, that is, the amplitude increases and the latency decreases. Different types of stimuli also lead to different results; and the result within the ASD group itself is different from that between ASD and the control group (Brandwein et al., 2015).

First, as for N1&P1, studies have shown that the amplitude of N1b is related to the severity of ASD symptoms, for example, the more severe the symptoms of ASD, the smaller the amplitude of N1b (Brandwein et al., 2015). However, a unified conclusion has not been reached in the comparison of P1&N1 waves between the ASD group and the control group. For example, some researchers believe that the N1b amplitude of the ASD group is smaller than that of the control group, which may be due to their insensitivity to sound, resulting in a smaller ERP amplitude (Bruneau et al., 1999); there are also results showing that the N1b amplitude of the ASD group is higher than that of the control group, and the latency is reduced. It was caused by the oversensitivity of the ASD group to sound stimuli (Oades et al., 1988). Both conclusions make sense somehow. In addition, it has been shown in the literature that the type of auditory stimulus also affects the amplitude of early auditory components in children with ASD. For example, ASD children will not have an increased P1 amplitude under the stimulation of exaggerated verbal stimuli, while normal children tend to increase significantly. It may explain that ASD children lack the neural reinforcement of verbal grandiosity (Chen et al., 2021). This may also explain the inability of children with ASD to understand some emotional words and sentences. But no studies have shown whether this phenomenon persists as children with ASD grow up.

Second, as for the MMN wave, some literature has shown that ASD children respond poorly to changes in some emotional stimuli (such as fear), so they have reduced MMN wave amplitude and prolonged latency to fearful sound stimuli (Korpilahti et al., 2007; Yoshimura et al., 2018). However, the researchers also did not get a unified conclusion on the amplitude and latency of MMN. Another literature believes that the MMN latency of ASD patients is smaller, and the amplitude is larger, indicating that ASD patients are more sensitive to differential stimuli (Gomot et al., 2002; Ferri et al., 2003). There is also literature showing that there is no significant difference in the MMN amplitude of ASD and the control group (Ceponiene et al., 2003). Like the auditory early component, it has been shown that stimulus type also plays a role in the properties of the MMN. For example, MMN waves disappear when ASD children change consonants, suggesting that ASD children have abnormal insensitivity to consonants (Lepistö et al., 2005).

Third, as for the P3 wave (which reflects a shift toward stimuli that requires a change in attention), some results show that there is no change in P3a amplitude in adults with ASD but there is an increased P3a amplitude in children with ASD (Gomot et al., 2002; Ferri et al., 2003), other results show that there is an increased P3a amplitude in adults (Iwanami et al., 2014). It seems that the amplitude of P3a is related to the subject’s age. Also, the type of stimulus is equally important. ASD children will only have a disappearance of P3a towards verbal stimuli, and they will not have a disappearance of P3a towards non-verbal stimuli (Ceponiene et al., 2003; Lepistö et al., 2005). This suggests that deficits in children with ASD occur when verbal attention is diverted. In addition, some literature pointed out that the two parameters dP3a and fP3a in P3a should be analyzed separately. The dP3a latency of the ASD group was shorter than that of the control group, and the more severe the symptoms of ASD, the shorter the dP3a latency. And only in the ASD group, the latency of fP3a becomes smaller with age, and there is no such trend in the control group; the more severe the symptoms of the ASD group (such as rejection of physical contact, etc.), the smaller the latency of fP3a (Chien et al., 2018). It is certain that ASD severity seems to be related to the latency of P3a reduction.

Finally, as for the N170 wave, the range of subjects discussed in current papers is wide: from ASD patients, ASD + TSC (tuberous sclerosis), ASD + ADHD, to family members of ASD children. At present, the generally accepted conclusions are: (1) ASD patients have poor ability to process faces. Most of the literature points out that the N170 latency of the control group was shorter when processing faces than when processing objects, but there was no significant difference between the processing of faces and objects in ASD children (Tye et al., 2015). The latency of N170 when processing faces in ASD patients is longer than that in the control group, and 6 literatures have reached this conclusion (McPartland et al., 2004; Webb et al., 2006; O'Connor et al., 2007; McPartland et al., 2011; Jeste et al., 2013; Tye et al., 2013); (2) 12 literatures point out that control group is more sensitive to upside-down faces than positive faces, and the latency of N170 is larger when observing upside-down faces, while ASD patients had no significant difference in N170 latency between upside-down and positive faces (Dawson et al., 2002; McPartland et al., 2004; Dawson et al., 2005; O'Connor et al., 2005, 2007; Webb et al., 2006; McCleery et al., 2009; Batty et al., 2011; Hileman et al., 2011; McPartland et al., 2011; Webb et al., 2012; Tye et al., 2015); in addition, for the lateralization of brain processing faces, 8 literatures believed that normal subjects’ N170 is right-sided (reflected in larger amplitude and shorter latency in the right hemisphere), by contrast, ASD patients’ N170 is left-sided or no significant difference between two hemispheres (Schultz et al., 2000; Pierce et al., 2001; Carver and Dawson, 2002; McPartland et al., 2004; Senju et al., 2005; McCleery et al., 2009; Tye et al., 2013, 2015); (3) ASD patients also have abnormal eye direction. Some studies suggest that the N170 latency of ASD to averted gaze is longer than that of direct gaze, and the processing of direct gaze is faster, while the control group has no significant difference between the two kinds of gazes (Senju et al., 2005). Some other literature pointed out that there is no significant difference between averted gaze and direct gaze in the ASD group. While the control group process direct gaze much faster. An interesting phenomenon is that the parents of ASD children do not seem to show the effect of left hemisphere lateralization in facial expression processing. While their N170 amplitude is larger in their right hemisphere than in their left hemisphere (Márquez et al., 2019).

### 3.2. Resting-state EEG abnormalities in ASD and its connection to attention diversion and memory

First, the literature shows that there is no significant difference between the ASD group and the control group in the resting EEG with eyes-closed conditions (Mathewson et al., 2012); however, when the eyes are open and there is visual stimulation, there are some differences in the power and coherence of delta, theta, beta, gamma, alpha in the ASD group. There are 9 literatures that show that the delta, theta, beta, gamma energy of ASD patients is higher than that of the control group, and the alpha energy is lower than that of the control group (Chan et al., 2007; Klimesch et al., 2007; Murias et al., 2007; Coben et al., 2008; Chan et al., 2009; Wang et al., 2013; Mably and Colgin, 2018; Brito et al., 2019; Neuhaus et al., 2021). And there are also 3 literatures showing that the alpha energy of the ASD group is higher than that of the control group (Cantor et al., 1986; Dawson et al., 1995; Mathewson et al., 2012). Because coherence and power are positively correlated, and phase synchronization is closely related to one’s ability to prepare for upcoming behaviors. For example, a lower alpha energy could explain a weaker ability to prepare for future behavior due to a lack in spike frequency or insufficient precision for ASD patients (Mathewson et al., 2012; Guyon et al., 2021).

Second, the degree of ASD symptoms is also a factor. Studies have shown that the more severe the ASD, the smaller the energy of gamma, delta, theta, and alpha, which is somewhat different from the above conclusions (Maxwell et al., 2015; Shephard et al., 2018). Gender is also a factor. As men grow older, the gamma energy decreases; and the stronger the social interaction ability of men with ASD, the lower theta, and alpha energies, but there is no such trend for women (Mathewson et al., 2012). Compared with the control group, the alpha frequency decreased more rapidly with age in the ASD group, which also seems to explain the faster loss of ASD’s ability to shift attention (Dickinson et al., 2022).

Finally, about long-range connectivity, only two literature believed that the temporal and frontal lobes connections of ASD were enhanced (Courchesne and Pierce, 2005; Murias et al., 2007), 17 papers believed that the ASD brain connection was weakened (Castelli et al., 2002; Belmonte et al., 2004; Just et al., 2004; Courchesne and Pierce, 2005; Villalobos et al., 2005; Cavanna and Trimble, 2006; Mottron et al., 2006; Boly et al., 2007; Just et al., 2007; Coben et al., 2008; Ben-Sasson et al., 2009; Weng et al., 2010; Marco et al., 2011; Cheng et al., 2015; Padmanabhan et al., 2017; Martínez et al., 2020; Wantzen et al., 2022). The scopes of insufficient connection involve frontal and bilateral temporal & occipital regions; and some higher-order regions which are related with neuron aging processes and pre-existing neuropathology; their default mode network (DMN), the sensorimotor network (SMN), the dorsal attention network (DAN) internal and inter-connection are also insufficient (Cheng et al., 2015; Padmanabhan et al., 2017; Wantzen et al., 2022). These deficiencies lead to memory loss, language deficits, decreased perception of environmental stimuli, and reduced ability to shift attention in ASD patients, which are some common behavioral symptoms in ASD subjects.

### 3.3. Magnetoencephalography markers

#### 3.3.1. Introduction to MEG and its advantages in measuring neural activity

Since MEG has ability to extract detailed information on the phase and frequency of neural and relative to EEG, MEG has a high temporal resolution and moderate spatial resolution responses, some research literature uses MEG to study the power band of different frequencies both under resting state and task states in patients with ASD. Functional connectivity and complexity in patients with ASD were also studied. Among the selected articles, a total of 8 discussed the application of MEG in ASD, two of which were measured under resting state and the other six were measured under task state.

#### 3.3.2. MEG findings related to sensory processing in ASD

There are 4 literature which point out that ASD children have an abnormal visual processing pattern as well as right lateralization. Besides, all of them study gamma band. Because of different tasks, these literatures draw different conclusions of gamma response. One study of visual tasks (Kikuchi et al., 2013) show that ASD children have a significant rightward connectivity between parietotemporal areas, which is also pointed out by another two reports (Koshino et al., 2005; Monk et al., 2009), *via* an excess of gamma band oscillation (Orekhova et al., 2007). It indicates that ASD children have an abnormal cortical information processing pattern during visual perception and attention (Jensen et al., 2007; Wang, 2010; Kikuchi et al., 2013). Another non-verbal visual reasoning task also achieved similar conclusions (Takesaki et al., 2016). This study shows that some of ASD patients have a better performance in visual reasoning tasks, because they have an increased connectivity with the visual area/stronger connectivity from the occipital area/increased gamma synchronization in V1 supragranular layers and influences V4 through feedforward projections (Khan et al., 2015), and there is a right lateralization (Kikuchi et al., 2013). It shows that the magnitude of feedforward connectivity associated with visual information represents a neurophysiological index of autistic visual strengths (Grandin, 2009a,b).

Two other experiments on visual processing concluded that ASD children’s gamma power was smaller in emotional processing and maternal face processing. One used the mother’s face to compare with non-facial stimuli (Hasegawa et al., 2023), showing that when ASD children look at their mother’s face, their low-frequency (30–59 Hz) gamma power in the right banks of superior temporal sulcus, right fusiform gyrus, right pericalrine cortex decrease compared to TD group; their high frequency (61–90 Hz) gamma power in right banks of superior temporal sulcus, bilateral fusiform gyrus and bilateral pericalcarine cortex also decrease compared to TD group, also revealing the right-sided gamma anomaly in children with ASD and its problems in social communication and face-processing. Another study concludes that young people with ASD have increased response times when looking at emotional faces, The intrinsic mechanism is that gamma responses from right occipital cortex to occipital-fusiform areas and occipital pole is largely absent (Bentin et al., 1996; Bailey et al., 2005; Wright et al., 2012). The conclusion may reveal a potential mechanism that may explain difficulties in face and emotion processing in ASD.

For auditory abnormalities of ASD hearing, studies have shown that the latency of M100 increases, and the more severe the ASD symptoms, the longer the latency of M100 is (100 ms is bilateral primary/secondary auditory cortex time duration). Besides, the transient gamma-band evoked power of ASD children decreases. It shows that ASD children have a perturbed auditory cortex neural activity/reduced conduction velocity (Gage et al., 2003; Wilson et al., 2007; Edgar et al., 2015a,b; Port et al., 2016). As for the factors of hemispheric laterality and age, another study showed that M100 was significantly delayed in the right hemisphere of ASD, and only the normal group had a decrease in M100 latency with increasing age, and ASD did not show this trend (Roberts et al., 2010). Only one study suggested that the M100 latency of ASD was smaller than that of TD (Ferri et al., 2003).

#### 3.3.3. MEG -based connectivity studies in ASD

**Figure 1:**
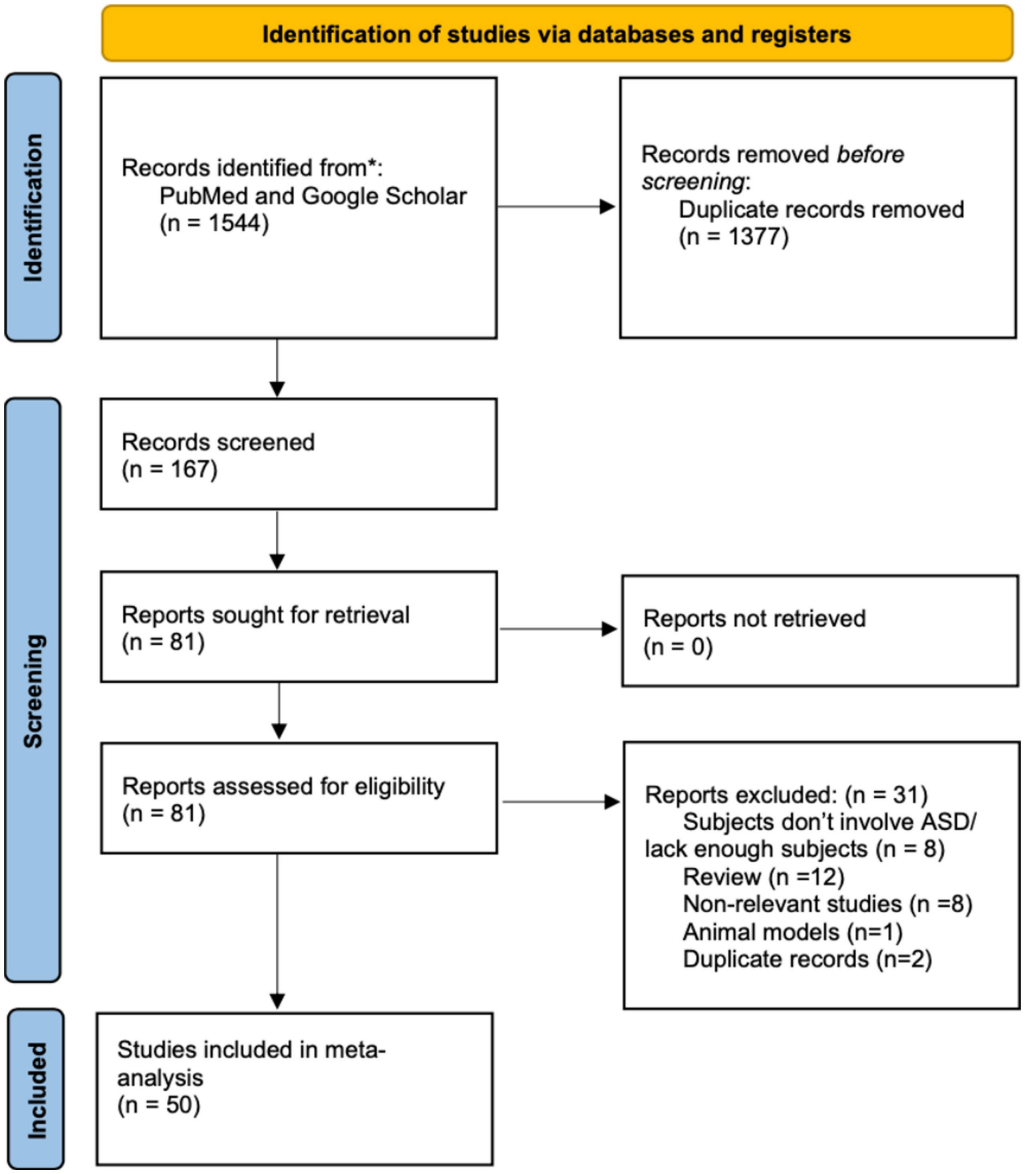
The screening process through this scoping review.

In an experiment of resting state activity, the functional connectivity (also the coherence between brain regions) of ASD and its complexity surprisingly compensate for each other, with one being higher and the other lower. ASD has a lower complexity in frontal regions in the delta band and occipital-parietal regions in alpha band and a higher complexity in parietal regions in the delta band, central and temporal regions in theta band, frontal-central boundary regions in the gamma band (Khan et al., 2013; Ghanbari et al., 2015). Comparatively, ASD has an increased short-range connectivity in frontal lobe in the delta band and increased long-range connectivity in temporal, parietal and occipital lobes in alpha band (Courchesne and Pierce, 2005; Barttfeld et al., 2011; Ghanbari et al., 2015). This is similar to the conclusion of another study that also studied resting state (Cornew et al., 2012). ASD’s relative delta power increases at frontal regions (Cantor et al., 1986; Murias et al., 2007) and alpha band power increases at temporal and parietal regions. In addition, results show that ASD has increased power of all delta and alpha band, theta band, beta and gamma band power (Orekhova et al., 2007; Cornew et al., 2012). Although a few studies believed that the alpha band power of ASD decreased (Cantor et al., 1986; Murias et al., 2007), this may be related to the fact that the experiment was done in the opening-eye state. This suggests that resting-state oscillatory activity in ASD is location-specific and supports the conclusion that connectivity in these regions increases (Figure 1).

### 3.4. Electrodermal activity markers

Overview of EDA as a measure of sympathetic nervous system activity and its application in ASD research. Electrodermal activity (EDA) is a property of our human bodies which causes continuous variation in the electrical characteristics of the skin. Our skin resistance varies with the state of sweat glands which is controlled by the sympathetic nervous system. The skin conductance is related with psychological or physiological arousal. If the sympathetic branch in the autonomic nervous system is aroused, the sweat gland activity increases, the skin conductance also increases. So currently, it is mostly used in clinic to track ASD children’s both psychologically and physiologically induced autonomic changes. It shows that ASD children have an abnormal pattern of EDA as well as a reduced average EDA in ASD children’s resting autonomic regulation. Also, evidence shows that there is a relationship between EDA and sensory symptoms or emotional dysregulation like anxiety as well as some repetitive behaviors in ASD children.

#### 3.4.1. Relationship between EDA markers and emotional regulation difficulties in ASD

The feasibility of using autonomic nervous system (ANS) activity as a marker of anxiety in ASD was explored in a study. Both typically developing children and children with ASD were examined, and significant changes in heart rate and electrodermal activity were observed during anxiety-inducing tasks. However, a differential pattern of response was found between the two groups, indicating an atypical autonomic response to anxiety in ASD characterized by sympathetic over-arousal and parasympathetic under-arousal. Variability in sympathetic nervous system arousal was further examined in relation to symptom severity in children with ASD. The study revealed that EDA in high-anxiety ASD group is different from low-anxiety ASD group. Low-anxiety ASD group has a relatively higher arousal (elevated EDA magnitudes, faster latencies, slower habituation) and high-anxiety ASD group has a lower arousal (lower EDA magnitudes, slower latencies, faster habituation; Panju et al., 2015).

Additionally, the relationship between EDA, sensory symptoms, and repetitive behaviors in children with ASD was explored. Although parents reported higher levels of sensory symptoms and repetitive behaviors in their children with ASD, no significant differences in EDA measures were found between the ASD and typically developing groups. This indicates that the reported differences in symptoms may not be directly related to measured EDA arousal or reactivity (McCormick et al., 2014).

Finally, a study examined the changes in skin conductance level (ΔSCL) in toddlers with ASD and typically developing toddlers in response to anger, joy, and fear emotions. Toddlers with ASD exhibited attenuated ΔSCL in the fear condition, which may predict the emergence of internalizing and externalizing problems. The study suggests that ΔSCL can serve as a dimension associated with behavioral responses in negatively emotionally challenging events in young children (Vernetti et al., 2020). In conclusion, the reviewed studies provide evidence for atypical autonomic function in ASD, particularly in sympathetic activity. The findings underscore the heterogeneity within ASD and emphasize the role of anxiety, autonomic features, and individual variability in understanding the autism spectrum. EDA shows promise as a potential measure of physiological arousal, anxiety, and individual differences within ASD, although further research is needed to fully elucidate its clinical utility.

### 3.5. Eye-tracking markers

#### 3.5.1. Importance of eye-tracking technology in studying social attention in ASD

Eye-tracking technology is the process of measuring either the point of gaze (eye positions) or the motion of an eye relative to the head (eye movement). Because eye-tracking technology can be used in both static tasks as well as dynamic tasks with videos and study abnormal ASD patients’ gaze pattern. Density including response time, fixation frequency, fixation duration, saccade amplitude can be studied in ASD group versus TD group. Several reviewed studies explain the potential relationship between ASD’s abnormal gazing pattern and their deficits in social attention and social motivation.

#### 3.5.2. Eye-tracking findings in individuals with ASD

ASD patients have failure to develop normal social relationships, and they also have sensory-perceptual processing deficits that weaken their abilities to attend and perceive social stimuli in daily living contexts. These behavioral abnormalities have something to do with their deficits in interpreting dynamic and interactive social stimuli, especially in reduced gaze at the organs like eyes and mouth. They are less sensitive to their motherese which is opposite in TD group. ASD children have a central coherence weakness (CWW) as well as worse gaze shift in joint attention following others, which means they focus more on specific things instead of global social context as well as they fail to focus on their attention as normal people do.

#### 3.5.3. Correlation between eye-tracking markers and social-communicative deficits in ASD

Different stimuli are studied in a study to test whether there is a significant difference between ASD group and TD group (Chevallier et al., 2015). Result shows that unlike static visual exploration task and dynamic visual exploration (faces and objects presented side-by-side), ASD children show a much less fixation time in the interactive visual exploration task (children are playing with objects) compared to TD group, which also indicates that ASD children have deficits in social attention because they have an abnormal gazing pattern in daily dynamic social stimuli depicting interaction.

Besides, in another study which explores the parts of face ASD patients prefer to gaze at (Jiang et al., 2019), ASD patients are relatively more sensitive to forehead, hair, ears, and chin which are irrelevant to emotion, and they are less sensitive to eyes and mouth compared to TD group. In general, ASD patients have a longer response time, fixation number (the number of fixations subjects make in each trial) and fixation duration, while they have a shorter in fixation frequency (the average number of fixation subjects make in each second of trial). This explains why ASD have difficulties in understanding others’ emotions and non-verbal communication.

Additionally, combining with results in fMRI, ASD children have a decreased eye-tracking related attention motherese with reduced activation in superior temporal area. However, TD group has the strongest response to motherese compared to mild and moderate affect speech. This indicates that ASD children have deficits in close relationship. Several studies propose mechanisms behind ASD patients’ deficits in social attention (Xiao et al., 2022). First one is CWW. This study analyzes ASD patients’ gazing pattern including what part of image and how long they gaze at, as well as let ASD patients to verbally report what they see on the screen. Results show that ASD children have more fixation number in localized AOIs instead of global picture. They cannot understand the whole picture of social context, failing to integrate social cues arising from the recognition of emotions in faces or from the environment in order to understand people’s interactions and relationship between social stimuli (Tsang and Chu, 2018; Tassini et al., 2022). Second one is both delay in response and shorter fixation time to visual attention to social stimuli, suggesting ASD patients may misinterpret social information and subsequent social cognitive processing because of skipping registering important momentary social information (Tsang and Chu, 2018). The third one is a reduced ability to engage in joint attention. ASD patients have less gaze shifts and lower gaze accuracy following others’ attention. And the more severe ASD symptoms are, the less the gaze shifts are, and lower gaze accuracy is. ASD patients’ gaze accuracy is lower when only eye gaze information is available than both eye gaze and head movement are available. This also shows that ASD children have difficulties in communication and social cognition (de Belen et al., 2023).

## 4. Conclusion

### 4.1. Recap of physiological biomarkers in ASD

In our review, we screened over 160 literatures, and we focused on 50 literatures in details. Through EEG, MEG, EDA, and eye-tracking, we conducted a complete and accurate review of ASD patients’ abnormal physiological biomarkers as well as their relationship with abnormal social behaviors. In general, ASD patients have abnormal latency, amplitude and power of EEG and MEG wave, suggesting they have abnormal sensory processing, and they also have an abnormal functional connectivity and complexity. Besides, ASD patients have an abnormal EDA and sympathetic nervous system activity, with higher possibility to have emotional regulation difficulties. ASD patients also have deficits in social attention with abnormal gazing pattern to faces and interpreting social context.

### 4.2. Potential applications and implications of these biomarkers in diagnosis and intervention

These biomarkers imply the abnormalities in social interaction, emotion, sensory processing in ASD patients’ daily life, which are what we expect. Through physiological biomarkers, researchers can find a bridge between neural abnormalities and behavioral deficits. For example, a low EDA level shows that patients have a high level of anxiety compared to the typical subjects; less fixation time to the interactive visual exploration tasks shows that patients have deficits in interaction and social communication; and a reduced MMN amplitude and a prolonged MMN latency show that patients have deficits in perceiving emotions. The potential applications of physiological biomarkers in ASD have bright future. For example, researchers can use these physiological biomarkers to detect early symptoms of ASD in children and do some interventions towards ASD. For example, early symptoms may include: no difference of N170 latency to upright and inverted faces; a lower complexity in frontal regions in the delta band and occipital-parietal regions in alpha band and a higher complexity in parietal regions in the delta band, central and temporal regions in theta band, frontal-central boundary regions in the gamma band, etc. Besides, researchers can use certain level of abnormalities in physiological biomarkers to grade the severity of ASD. For instance, the more severe ASD is, the smaller the latency of P3a is; the more severe the symptoms of ASD are, the smaller the amplitude of N1b is; and the more severe the ASD, the smaller the energy of gamma, delta, theta, and alpha are, etc.

### 4.3. Future directions for research and advancements in the field

Future research should focus more on the studies about EDA and eye-tracking because there are not so many pieces of records on these topics, which means that single and separate result may not ensure the generalizability of the conclusion. Besides, more unified results about EEG and MEG should be made because currently, results really diverge on the amplitude and latency of magnetoencephalogram and electroencephalogram, increasing the difficulty of recognizing and treating ASD patients. More accurate devices, more rigorous measuring methods, and more subjects should be considered in the future studies.

## 5. Limitation

In this research, we did not include the studies of fNIRS and fMRI as our first proposed title is “A Scoping Review of Electrophysiological Markers in Autism.” However, it will be complete to also study what have been done for fNIRS and fMRI. Besides, because we initially wanted to do a systematic review with all literatures in this field, we failed to focus on the novelty of studies, especially for the studies in latest 3 years. In the future, we will include more recent study results. Last but not least, we will modify our review format into a systematic review and do relative meta-analysis to provide a more complete and effective review in the future.

## Author contributions

JS: Conceptualization, Formal analysis, Investigation, Methodology, Validation, Visualization, Writing – original draft, Writing – review & editing. YG: Conceptualization, Formal analysis, Investigation, Methodology, Validation, Visualization, Writing – original draft, Writing – review & editing. JZ: Conceptualization, Formal analysis, Investigation, Methodology, Validation, Visualization, Writing – original draft, Writing – review & editing. XH: Conceptualization, Formal analysis, Investigation, Validation, Visualization, Writing – original draft, Writing – review & editing. HW: Conceptualization, Formal analysis, Investigation, Resources, Validation, Writing – original draft, Writing – review & editing. TY: Conceptualization, Formal analysis, Investigation, Validation, Visualization, Writing – original draft, Writing – review & editing. DZ: Conceptualization, Formal analysis, Investigation, Supervision, Validation, Visualization, Writing – original draft, Writing – review & editing.

## Funding

The author(s) declare financial support was received for the research, authorship, and/or publication of this article. This study was supported by National Science and Technology Innovation 2030 Major Project of China (2021ZD0203900), NSFC grants (81822017, 82271530, 32241015, 31900765), the Lingang Laboratory (grant no. LG-QS-202203-10), the Science and Technology Commission of Shanghai Municipality (23ZR1480800, 22QA1407900, 21YF1439700), Shanghai Municipal Commission of Health (2022JC016), Shanghai Municipal Education Commission - Gaofeng Clinical Medicine Grant Support (20181715), Innovation teams of high-level universities in Shanghai, Project of Sichuan Department of Science and Technology (grant no. 2023YFS0267, 2022NSFSC1374, and 2021YFS0385), and Project of Sichuan Provincial People’s Hospital (grant no. 2022QN04).

## Conflict of interest

The authors declare that the research was conducted in the absence of any commercial or financial relationships that could be construed as a potential conflict of interest.

## Publisher’s note

All claims expressed in this article are solely those of the authors and do not necessarily represent those of their affiliated organizations, or those of the publisher, the editors and the reviewers. Any product that may be evaluated in this article, or claim that may be made by its manufacturer, is not guaranteed or endorsed by the publisher.
